# Research on safety condition assessment methodology for single tower steel box girder suspension bridges over the sea based on improved AHP-fuzzy comprehensive evaluation

**DOI:** 10.1038/s41598-024-61579-1

**Published:** 2024-05-27

**Authors:** Huifeng Su, Cheng Guo, Ziyi Wang, Tao Han, David Bonfils Kamanda, Fengzhao Su, Liuhong Shang

**Affiliations:** 1 College of Transportation, Shandong University of Science and Technology, Qingdao, 266590 China; 2 Key Laboratory of Transportation Infrastructure Performance and Safety in Shandong Province Universities, Qingdao, 266590 China; 3 Shandong Road and Bridge Group Co., Ltd., Qingdao Branch, Qingdao, 266100 China; 4 Shandong Expressway Qingdao Development Co., Ltd., Qingdao, 266000 China

**Keywords:** Single tower steel box girder suspension bridges over sea, Health monitoring, Improved Analytic Hierarchy Process (AHP), Fuzzy comprehensive evaluation method, Safety assessment, Engineering, Mathematics and computing

## Abstract

In order to propose a reliable method for assessing the safety condition for single-tower steel box girder Suspension bridges over the sea, a condition monitoring system is established by installing sensors on the bridge structure. The system is capable of gathering monitoring data that influence the safety status of the bridge. These include cable tension, load on the main tower and pylon, bearing displacement, wind direction, wind speed, and ambient temperature and humidity. Furthermore, an improved Analytic Hierarchy Process (AHP) algorithm is developed by integrating a hybrid triangular fuzzy number logic structure. This improvement, coupled with comprehensive fuzzy evaluation methods, improves the consistency, weight determination, and security evaluation capabilities of the AHP algorithm. Finally, taking the No.2 Channel Bridge as an example and based on the data collected by the health monitoring system, the application of the safety assessment method proposed in this paper provides favorable results in evaluating the overall safety status of the bridge in practical engineering applications. This provides a basis for management decisions by bridge maintenance departments. This project confirms that the research results can provide a reliable method for assessing the security status of relevant areas.

## Introduction

With the in-depth implementation of the strategy to strengthen national transport, the development of transport infrastructure has entered a new phase of rapid development. It is expected that China could lead the world in the number of bridges by the 2030s^1^. As the service life of bridges increases, damage to various structures and components can have an impact on the safe operation of bridges. In some cases, the failure of a particular component can result in a complete loss of bridge safety. In order to be able to assess the safety status of bridges intuitively and quickly, it is usually necessary to carry out safety assessments. There are two main methods for assessing bridge safety: using bridge monitoring data and using manual inspections along with standardized criteria. Currently, the assessment and early warning of the safety status of bridge structures is largely carried out by installing sensors and monitoring devices on bridge structures. This enables long-term real-time monitoring of the operating status and relevant physical parameters of the bridge^2,3^. For single-tower steel box girder suspension bridges over the sea, traditional manual inspection methods suffer from subjectivity, low efficiency and high labor costs due to their high pylons and structural complexity, so they cannot meet maintenance requirements. Therefore, in order to capture the safety operation status of bridge structures in real time, it is particularly important to conduct safety status assessments for single-tower steel box girder suspension bridges over the sea using health monitoring systems.

Bao et al.^4^ In order to carry out an effective risk assessment in the construction of long-span bridges and determine the optimal construction scheme using the Analytic Hierarchy Process, the AHP was integrated with the Gray Correlation method. They created a multi-level comprehensive assessment model and used the AHP to provide weights for the factors that influence the assessment indicators. Yang et al.^5^ based on a comprehensive analysis of the safety factors associated with existing bridges crossing municipal roads, proposed a comprehensive fuzzy evaluation method of Analytic Hierarchy Process to evaluate the impact of road construction on the safety of existing bridges. Yang et al.^6^ proposed a novel comprehensive condition assessment method that considers the uncertainty of the measured data intervals and the influence of conflicting measured data. By comparing the condition assessment results with the actual state of components or the entire bridge, they verified the advantages of the proposed method over existing AHP assessment methods and traditional combination methods. Liu et al.^7^ presented a reliability assessment method for a precast reinforced concrete hollow slab bridge system considering damage to joint nodes based on an improved Analytic Hierarchy Process. Tan et al.^8^ addressed the optimization selection problem of retrofit solutions for old bridges and introduced a decision method based on fuzzy Analytic Hierarchy Process weights and gray relational analysis. Lu et al.^9^ proposed a method for risk assessment of Suspension bridges and cable systems based on cloud model, which effectively combines the randomness and uncertainty of risk information. Wang et al.^10,11^ outlined the research trends in main cable safety assessment and emphasized the importance of improving the safety of main cables to ensure the structural safety of long-span, multi-tower suspension bridges. Andrić et al.^12^ combined the Fuzzy Analytic Hierarchy Process (FAHP) with fuzzy knowledge representation and fuzzy logic techniques, proposing a novel framework for disaster risk assessment. This method proves its practicality and efficiency in analyzing and evaluating multi-hazard risks for bridges. Ji et al.^13^ introduced a large-scale risk assessment method for complex bridge structures based on Delphi-enhanced Fuzzy Analytic Hierarchy Process (FAHP) factor analysis. The approach was validated through a comparative study with practical engineering cases and the Analytic Hierarchy Process, confirming its feasibility and practicality. It serves as a reference for later risk prevention in bridge. Liang^14^ presented a multi-level evaluation system suitable for assessing the health status of prestressed continuous concrete bridges. This innovative rating system effectively supports bridge management and maintenance. Deng et al.^15^ developed a comprehensive assessment method for the safety and reliability of existing railway bridges. The method serves as a theoretical basis for the maintenance and strengthening of the Songhua River Bridge on the Binbei Line. Ma et al.^16^ proposed a systematic safety assessment for overwater bridge transportation, a technology that significantly increases the safety of bridges during sea transportation. Maljaars et al.^17^ developed an evaluation method to determine the actual safety level of highway bridges and viaducts. This method focuses on assessing the impact of traffic behavior and consists of several levels. Zhu et al.^18^ conducted an in-depth study on the safety assessment methods for Bridge Health Monitoring Systems (BHMS) using comprehensive fuzzy assessment techniques. They developed a novel Bridge Health Monitoring System based on safety assessment vectors. Li et al.^19^ introduced a new security assessment method that combines Monte Carlo simulation (MCS) and Bayesian theory. This method enables reliable assessment and back-diagnosis of the overall safety performance of reinforced concrete bridges in cold regions. Fu et al.^20^ used multi-source data from the construction and dismantling of a large-span reinforced concrete arch bridge in China. They applied the Analytic Hierarchy Process AHP to analyze the data from multiple sources and set a safety alarm threshold for the bridge during construction. Miyamoto et al.^21^ proposed an early warning method for bridge safety using wireless sensor network technology. The method showed satisfactory results in various performance indicators such as flood delay, energy efficiency and throughput. Li et al.^22^ established a risk assessment index system for safety in the operational phase of highway bridges. They then used cloud entropy weighting to objectively weigh various risk indicators and applied cloud model theory to risk assessment, emphasizing the objectivity of the assigned values. Feng et al.^23^ presented an innovative approach that combines the Analytic Hierarchy Process (AHP) with the Finite Element Method (FEM). This approach highlighted the potential risk of influence of uncertain factors on the environment. Li et al.^24^ proposed a probabilistic performance evaluation framework for a Suspension bridge, which considers factors such as wind speed, wind direction, bridge orientation, wind-wave correlation and parameter uncertainty. This framework provides a comprehensive and practical method for evaluating the performance and optimizing the design of SCBs under wind and wave loads. Xu et al.^25^ presented a cloud-based Analytic Hierarchy Process (C-AHP) scoring system for determining inspection intervals. The proposed C-AHP rating system not only takes into account the vagueness of the AHP rating system, but also addresses its randomness and provides more stringent time intervals for routine inspections of long-span suspension bridges compared to the F-AHP rating system. Prasetyo et al.^26^ used AHP and Promethee II methods to analyze and prioritize the ideal weight criteria for bridge handling. This approach makes the priority weighting process more dynamic and manageable.

In summary, there exists a paucity of research both domestically and internationally concerning the safety assessment of single tower suspension bridges featuring a steel box girder structure spanning over open sea expanses. In the field of safety assessment analysis for bridge structures, the traditional AHP is commonly used. In the traditional AHP framework, assessment matrices are created based on pairwise comparisons of selected criteria. However, the requirement for precise numerical values ​​within these matrices requires respondents to have a thorough understanding of the relative importance of each choice. In practice, due to the complexity of objective phenomena and the human mind's use of fuzzy concepts, describing relative importance with precise numbers (such as 3, 1/9, etc.) becomes challenging. This leads to low credibility of weight calculation, cumbersome calculations, and weakened ability to comprehensively evaluate. Further refinements and improvements are required to determine the weights and improve the scoring matrix in a more meaningful way. Given this background, the present study improves the judgment matrix through a hybrid triangular fuzzy number logic structure with the aim of accounting for the uncertainty inherent in human analysis and cognition. This extension includes specifying the upper and lower limits of the possibility intervals as well as the most likely central values. By using the membership function of triangular fuzzy numbers, the study derives the possibilities of various parameters within the entire interval range. This method improves the determination of weights in the AHP, thereby improving its consistency and weight solution capabilities. By combining the improved AHP method with comprehensive fuzzy evaluation, the study proposes an improved AHP-fuzzy comprehensive evaluation approach to evaluate the safety status of a single-tower steel box girder suspension bridge over the sea. This approach increases the accuracy and rationality of the assessment results and aims to address the shortcomings in the safety assessment research of such bridge structures and provide valuable insights for the safety assessment of bridges.

## Method for assessing the safety status of a single tower steel box girder suspension bridges over the sea

### Basic principles of improved analytic hierarchy process

The Analytic Hierarchy Process, introduced by American operations research professor Saaty in the 1970s^27^, is an effective method that converts semi-qualitative and semi-quantitative problems into quantitative calculations. AHP is known for its simplicity, rigorous mathematical foundation, and widespread application in analysis and decision making of complex systems. It serves as a practical, multi-criteria decision-making method and offers advantages such as systematicity, conciseness, flexibility and usefulness.

In traditional AHP, judgment matrices are determined through pairwise comparisons of selected criteria, which requires respondents to have a clear understanding of the relative importance of each selection. However, in practice, due to the large number of evaluation criteria in the AHP evaluation process, the complexity of objective phenomena and the application of fuzzy concepts in human thinking, experts find it difficult to give an accurate value when evaluating pairwise comparison indicators. Restricting the evaluation of importance levels to fixed and finite numbers ignores the fuzziness of experts' thought processes during evaluation, which leads to inconsistency problems in the evaluation matrices and to some extent limits the accuracy of the evaluations. To address this problem, this study integrates the triangular fuzzy number method, improves the weight determination method in the analytical hierarchy process, and improves its consistency and weight solution capabilities. Triangular fuzzy numbers represent a range concept that specifies the upper and lower limits of a probability interval as well as the maximum probability value. By using the membership function of triangular fuzzy numbers, the probabilities of various parameters within the entire interval range can be determined.

1. When constructing the judgment matrix $A = \left( {a_{ij} } \right)_{n \times n}$, we depart from the conventional method of using a single precise numerical value to represent the importance of two indicators, and instead employ the method of triangular fuzzy numbers to indicate the interrelationships between pairs of indicators. First, the most probable value “m” is determined, which represents the basic assessment of the relationship between the two indicators, followed by the establishment of the upper and lower limits, denoted “a” and “b”. The lower bound represents the minimum rating that experts consider possible, while the upper bound represents the maximum possible rating. Finally, an importance interval is provided, denoted as $a_{ij} = \left[ {a_{ij} ,m_{ij} ,b_{ij} } \right]$, where “$a$” represents the minimum importance value in the comparison of the two indicators, “$m$” denotes the most likely value in the comparison. and “$b$” denotes the maximum importance value in the comparison.
2. Using formula (1), transform the interval form of importance into specific precise numerical values-and obtain a consistent judgment matrix without the need for consistency checks.
  1 $$a_{ij} = \frac{a + 4m + b}{6}$$

Regarding formula (1), Professor Hua Luogeng has previously provided explanations for similar formulas: The probability of “$a_{ij}$” taking the minimum value, “$a$” and “$b$” taking the maximum value is relatively small, and the probability distribution closely follows the normal distribution distribution pattern. Therefore, assuming that “$a_{ij}$” takes the most likely value “$m$” is twice as likely as assuming that “$a$” takes the minimum value and “$b$” takes the maximum value. The weighted average algorithm produces the following results:

$$a_{ij} = \frac{1}{2} + \left( {\frac{a + 2m}{3} + \frac{b + 2m}{3}} \right) = \frac{a + 4m + b}{6}$$

For example, if an expert's assessment of the relative weights of Indicator 1 and Indicator 2 is (2/3, 1, 3/2), then that expert's assessment of the weight of Indicator 1 relative to Indicator 2 is:

$$a_{12} = \frac{{\frac{3}{2} + 4 \times 1 + \frac{2}{3}}}{6} = \frac{37}{{36}}$$

### Improving the basic steps of the analytic hierarchy process

1. Clearly define the basic problems and relevant influencing factors
  At the initial stage, it is important to have a comprehensive understanding of the problems being studied and the problems to be solved. The aim is to clearly identify the overarching problem, i.e. the end goal. After defining the basic problem, it is then a matter of identifying the relevant influencing factors that can play a role in solving the problem. These include both primary and secondary factors.
2. Establishing a hierarchical structure
  Establishing a hierarchical structure is a crucial step in the AHP, especially when assessing the comprehensive safety status of bridges. The initial phase involves systematically categorizing the research problem and organizing it into hierarchical layers and thus constructing an evaluative indicator system or model. Within this system or model, the research problem is delineated into different indicator elements at different levels. These indicator elements are further classified based on their unique properties. In particular, each set of indicators at a lower level should be subordinate to the indicators at the level above. To improve the overall rationality of the hierarchical system or model, the division of hierarchical levels should conform to principles such as security, simplicity, independence and objectivity. The hierarchical structure can basically be divided into three levels:
  1. Top level (target level): This level is also called the target level and contains only one indicator element. In the context of this document, the top level is the comprehensive safety assessment of the 2nd canal bridge.
  2. Intermediate level (criterion level): This level is also called the criterion level and can contain several indicator elements. Each indicator at this level is constrained by and subordinate to the top level indicator. The indicators at this level should share common attributes. For example, in the case of a suspension bridge, the central indicators may include components such as main beams, main tower, main cables, hanging rods, etc.
  3. Bottom level (alternative level): This level is also called alternative level and can also contain several indicator elements. These indicators represent various solution measures for achieving the goal. Each indicator at this level should have an influencing factor on the security status of the higher-level indicators. For example, within the “main beam” indicator at the middle level, the indicators at the lowest level could include the stress and displacement of the main beam. Stress and displacement can be further divided based on different locations and directions.
  An ideal typical analytic hierarchy model is shown in Fig. 1.

**Figure 1:**
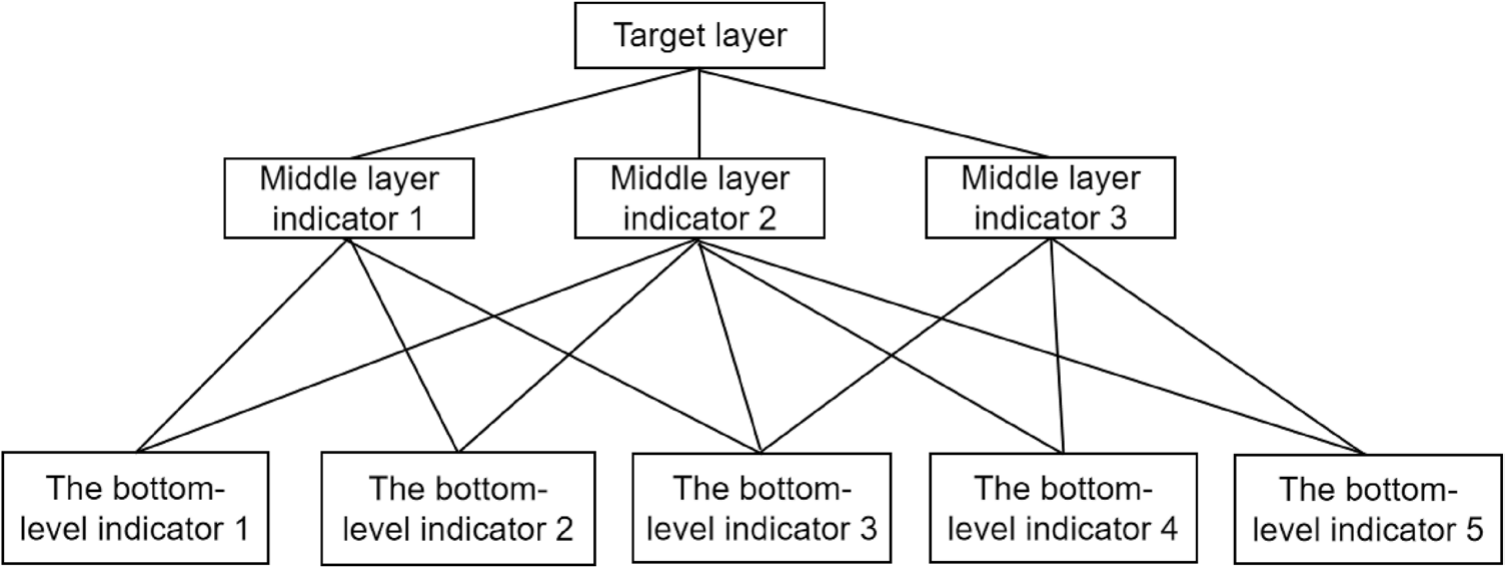
Ideal typical AHP model.
3. Construction of a triangular judgment matrix in fuzzy number form
  Within the same hierarchy, different indicator elements are categorized into multiple levels based on their respective excellence or importance. Quantitative values are assigned to represent these levels. If the precision requirements are low, a 5-step quantitative method can be used, using the integers 1, 3, 5, 7 and 9 to express the importance of one indicator element over another. This is called the 5-stage quantitative method, where a higher number indicates greater importance of the former over the latter. To express the former as less important than the latter, the reciprocal of 1, 3, 5, 7 and 9 can be used. If higher precision in level division is required, interpolation can be applied within the 5 level method by introducing 2, 4, 6 and 8, creating a 9 level quantitative method. The meaning of the scale from 1 to 9 is shown in Table 1.

**Table 1:** Judgment matrix scale.

Scale	Meaning
1	Two elements are of equal importance in comparison
3	In comparison, the former is slightly more important than the latter
5	In comparison, the former is significantly more important than the latter
7	In comparison, the former is strongly more important than the latter
9	In comparison, the former is extremely more important than the latter
2, 4, 6, 8	The intermediate value between the two adjacent judgments mentioned above
Reciprocal	Indicating the importance of comparing the interchangeability of the two corresponding factors
  Distribute evaluation matrix evaluation sheets to relevant experts and guide them to evaluate the scale table using the hierarchical analysis method described above. They are expected to perform pairwise comparisons of the indicators and then assign importance values. Summarize the assessments of each expert and create the evaluation matrix in the form of triangular fuzzy numbers, as shown in (2).
  2 $$B = \left[ {\begin{array}{*{20}c} {\left[ {1,1,1} \right]} & {\left[ {a_{12} ,m_{12} ,b_{12} } \right]} & \cdots & {\left[ {a_{1n} ,m_{1n} ,b_{1n} } \right]} \\ {\left[ {a_{21} ,m_{21} ,b_{21} } \right]} & {\left[ {1,1,1} \right]} & \cdots & {\left[ {a_{2n} ,m_{2n} ,b_{2n} } \right]} \\ \vdots & \vdots & \ddots & \vdots \\ {\left[ {a_{n1} ,m_{n1} ,b_{n1} } \right]} & {\left[ {a_{n2} ,m_{n2} ,b_{n2} } \right]} & \cdots & {\left[ {1,1,1} \right]} \\ \end{array} } \right]$$
  The approximation of the consistency of the matrix “A” leads to the generation of the consistency assessment matrix $M = \left( {m_{ij} } \right)_{n \times n}$, whereby the parameter $m_{ij}$ is calculated as follows:
  3 $$m_{ij} = \frac{{a_{ij} + 4m_{ij} + b_{ij} }}{6}$$
  $$M = \left[ {\begin{array}{*{20}c} {{\text{m}}_{{{11}}} } & {{\text{m}}_{{{12}}} } & \cdots & {{\text{m}}_{{{\text{1n}}}} } \\ {{\text{m}}_{{{21}}} } & {{\text{m}}_{{{22}}} } & \cdots & {{\text{m}}_{{{\text{2n}}}} } \\ \vdots & \vdots & \ddots & \vdots \\ {{\text{m}}_{{{\text{n1}}}} } & {{\text{m}}_{{{\text{n2}}}} } & \cdots & {{\text{m}}_{{{\text{nn}}}} } \\ \end{array} } \right] = \left[ {\begin{array}{*{20}c} {1} & {{\text{m}}_{{{12}}} } & \cdots & {{\text{m}}_{{{\text{1n}}}} } \\ {{\text{m}}_{{{21}}} } & {1} & \cdots & {{\text{m}}_{{{\text{2n}}}} } \\ \vdots & \vdots & \ddots & \vdots \\ {{\text{m}}_{{{\text{n1}}}} } & {{\text{m}}_{{{\text{n2}}}} } & \cdots & {1} \\ \end{array} } \right]$$
4. Based on the consistency judgment matrix, calculate the weights of each indicator
  ① Start by calculating the nth root of the product of the elements in each row of the consistency judgment matrix.
    4 $$\overline{W} = \sqrt {M_{i} }$$
    where $$M_{i} \prod\nolimits_{j = 1}^{n} {m_{ij} }, \;\; \left( {i = 1,2, \ldots n} \right)$$.
  ② Orthogonalize the above calculation results to obtain coefficients for each evaluation indicator.
    5 $$W_{i} = \frac{{\overline{{W_{i} }} }}{{\sum\nolimits_{j = 1}^{n} {w_{j} } }}$$
    $W_{i} = \left( {W_{1} ,W_{2} , \ldots ,W_{n} } \right)^{T}$ therefore denotes the weight coefficients determined for the respective evaluation indicators.

### Basic principle of the comprehensive fuzzy evaluation method

In 1965, Professor L.A. Zadeh from the United States published an article on fuzzy logic in an international journal in which he established the concept of fuzzy set theory and marked the birth of fuzzy mathematics^28^. Fuzzy or uncertain entities can be described using fuzzy mathematics. The term “fuzzy” refers to the variability between objective units that arises from the uncertainty in classifying units due to subjective differences. It is a form of description for concepts that are clearly defined but have unclear boundaries. In practical life, many concepts are vague, such as: youth, early morning, cold, etc. Due to subjective and objective limitations, each individual has different mental limits for these phenomena, which reflect people's subjective factors. When the fundamental concepts are unclear, accurate identification of an object is unrealistic. Instead, one can only assess the extent to which the object is likely to correspond to the concept.

#### Fuzzy sets and membership functions

In classical set theory, for a given element “${\text{x}}$”, its membership in the classical set “${\text{A}}$” is clear. The relationship between the two is binary, either belonging or not belonging, a clear distinction represented by either ${\text{x}} \in A$ or ${\text{x}} \notin A$. This relationship can be described using a characteristic function. However, for certain indefinite quantities or units, their values cannot be determined precisely. Therefore, it becomes necessary to apply fuzzy set theory to handle such cases.

In fuzzy set theory, the transformation of the characteristic function into a membership function is used to solve problems. Membership degrees are used to reflect the degree of membership of a fuzzy set to a fuzzy set. Assuming a discourse universe “${\text{U}}$” and a set “${\text{A}}$”, for each element ${\text{x}} \in A$, a function $\mu_{A} \left( {\text{x}} \right) \in \left[ {01} \right]$ can be used to represent the degree to which element “${\text{x}}$” belongs to the set “${\text{A}}$”, as follows:

6 $$\begin{gathered} \mu_{A} :U \to \left[ {0,1} \right] \hfill \\ {\text{x}} \to \mu_{A} \left( x \right) \hfill \\ \end{gathered}$$

In the context of fuzzy set theory, the range “${\text{U}}$” is called the set of elements, while the set “${\text{A}}$” is called a fuzzy set. The function $\mu_{A} \left( {\text{x}} \right)$, called the membership function, serves as the membership function for “A”. In this scenario, a fuzzy set can be fully represented by a corresponding fuzzy function. The membership function $\mu_{A} \left( {\text{x}} \right)$ assigns values ranging from 0 to 1, where the value is to 1, the higher the degree of membership of the element “${\text{x}}$” to a fuzzy set in the fuzzy set”${\text{A}}$”; the closer it is to 0, the lower the degree of membership of the element “${\text{x}}$” to a fuzzy quantity in the fuzzy set ”${\text{A}}$”.

#### Methods for determining membership functions

Fuzzy set and membership function are inextricably linked. The fuzzy set is represented by the membership function. The membership function is also the implementation of fuzzy set operations. Using the correct membership function is the basis for applying fuzzy set theory to solve practical problems. This article uses fuzzy statistics to determine the membership function.

Fuzzy statistics are used to represent the membership function in a similar way to probability statistics to determine the degree of membership. The basic steps are as follows: First, a fuzzy set “A” and a discourse area “U” are determined. Then, based on their personal experience, several experts or scientists judge which fuzzy set or which fuzzy evaluation interval of a specific element “${\text{x}}_{0}$” in the discourse area ”U” belongs to the fuzzy set “A” The expression of the membership function can be expressed as follows:

7 $$\mu \left( {{\text{x}}_{0} } \right) = \frac{{{\text{The number of times }}\text{``}{\text{x}}_{0} \in A\text{''}}}{{\text{n}}}$$

where “n” is the number of experts or scientists. In this way, the membership level is determined by the statistical membership frequency. When “n” experts are invited to an experiment, the membership frequency “$\mu$” tends to the stable value as the “n” value increases, and the stable frequency value is the membership degree of the element “${\text{x}}_{0}$” belonging to the fuzzy set “A”.

### Basic steps of first-level comprehensive fuzzy evaluation

1. Identification of the factor set
  When conducting fuzzy assessments, the first step is to identify the various factors that affect the target's assessment results. For example, in a comprehensive safety assessment of a suspension bridge, the influencing factors include the main girder, main tower, main cables, hanging rods and others. The totality of these individual factors is called a factor set and is usually denoted by the symbol “U”. This can be expressed as follows:
  8 $${\text{U}} = \left\{ {\mu_{{1}} ,\mu_{{2}} , \ldots ,\mu_{{\text{n}}} } \right\}$$
2. Determine the factor weight vector
  In the determined factor set ${\text{U}} = \left\{ {\mu_{{1}} ,\mu_{{2}} , \ldots ,\mu_{{\text{n}}} } \right\}$, each factor has a different influence on the evaluation goal. Therefore, it is necessary to meaningfully divide the weight of each factor and assign a corresponding weight value, which can be determined through an analytical hierarchy process. The weight value of each factor can be converted into a weight vector, generally expressed by “A”:
  9 $${\text{A}} = \left( {{\text{a}}_{{1}} ,{\text{a}}_{{2}} , \ldots ,{\text{a}}_{{\text{n}}} } \right)$$
  10 $$\sum\limits_{1}^{{\text{n}}} {{\text{a}}_{{\text{i}}} } = 1$$
  In the formula, $a_{1} ,a_{2} , \ldots a_{n}$ represents the weight value corresponding to the factor $u_{1} ,u_{2} , \ldots u_{n}$, and $0 \le a_{i} \le 1$.
3. Determine the amount of fuzzy comments
  After determining the factor set ${\text{U}} = \left\{ {\mu_{{1}} ,\mu_{{2}} , \ldots ,\mu_{{\text{n}}} } \right\}$, a corresponding fuzzy comment set needs to be created so that the evaluator can achieve specific judgment results for each element in the factor set. For example, according to the classification of the technical condition of a bridge, the bridge can be divided into categories 1, 2, 3, 4 and 5, and the corresponding fuzzy comments on the bridge status include intact, good, fairly good, poor and dangerous. The set of fuzzy evaluation is called fuzzy evaluation theorem and is generally used in the “V” representation, that is:
  11 $${\text{V}} = \left\{ {{\text{v}}_{{1}} ,{\text{v}}_{{2}} , \ldots ,{\text{v}}_{{\text{m}}} } \right\}$$
  In the equation, ${\text{v}}_{1} ,{\text{v}}_{2} , \ldots ,{\text{v}}_{m}$ represents “m” fuzzy evaluations created for each factor.
4. Single factor evaluation
  The single factor evaluation refers to the individual evaluation of each factor within the factor set “U”. This process determines the degree of membership of each factor to different ratings in the fuzzy rating set “V”. For example, when evaluating the “i”-th factor $\mu_{{\text{i}}}$ within the factor set “U”, the degree of membership of this factor to the “j”-th evaluation “V” in the fuzzy evaluation set ${\text{v}}_{{\text{j}}}$ can be specified as ${\text{r}}_{{{\text{ij}}}}$. The membership degrees obtained for the ${\text{i}}$th factor $\mu_{{\text{i}}}$ can be represented as $r_{j}$, which in the context of bridge building can be expressed as follows:
  12 $${\text{r}}_{{\text{i}}} = \left\{ {{\text{r}}_{{{\text{i1}}}} ,{\text{r}}_{{{\text{i2}}}} , \ldots ,{\text{r}}_{{{\text{im}}}} } \right\}$$
  In the equation, ${\text{r}}_{{{\text{i1}}}} ,{\text{r}}_{{{\text{i2}}}} , \ldots ,{\text{r}}_{{{\text{im}}}}$ represents the membership degrees of the ${\text{i}}$ th factor to ${\text{m}}$ fuzzy evaluations, where $0 \le {\text{r}}_{{{\text{im}}}} \le 1$.
5. Building a comprehensive fuzzy evaluation matrix
  When evaluating a goal with multiple influencing factors, the aggregation of the membership degree sets resulting from the evaluation of all factors within the factor set ${\text{U}}$ leads to the creation of a comprehensive assessment matrix for the evaluation goal. This matrix is usually represented by the symbol ${\text{R}}$. It can be expressed as:
  13 $${\text{R}} = \left[ {\begin{array}{*{20}c} {{\text{r}}_{{1}} } \\ {{\text{r}}_{{2}} } \\ \vdots \\ {{\text{r}}_{{\text{n}}} } \\ \end{array} } \right] = \left[ {\begin{array}{*{20}c} {{\text{r}}_{{{11}}} } & {{\text{r}}_{{{12}}} } & \cdots & {{\text{r}}_{{{\text{1m}}}} } \\ {{\text{r}}_{{{21}}} } & {{\text{r}}_{{{22}}} } & \cdots & {{\text{r}}_{{{\text{2m}}}} } \\ \vdots & \vdots & \ddots & \vdots \\ {{\text{r}}_{{{\text{n1}}}} } & {{\text{r}}_{{{\text{n2}}}} } & \cdots & {{\text{r}}_{{{\text{nm}}}} } \\ \end{array} } \right]$$
6. Fuzzy comprehensive evaluation
  After determining the weight vector $A_{1 \times n}$ for each factor and constructing the comprehensive judgment matrix $R_{{{\text{n}} \times {\text{m}}}}$, fuzzy transformation is applied to both using fuzzy operators. This process produces a fuzzy valuation vector ${\text{B}} = \left( {{\text{b}}_{{1}} ,{\text{b}}_{{2}} , \ldots ,{\text{b}}_{{\text{m}}} } \right)$, the calculation formula of which is expressed as follows:
  14 $$B = A \circ R = \left( {a_{1} ,a_{2} , \ldots ,a_{n} } \right) \circ \left[ {\begin{array}{*{20}c} {{\text{r}}_{{{11}}} } & {{\text{r}}_{{{12}}} } & \cdots & {{\text{r}}_{{{\text{1m}}}} } \\ {{\text{r}}_{{{21}}} } & {{\text{r}}_{{{22}}} } & \cdots & {{\text{r}}_{{{\text{2m}}}} } \\ \vdots & \vdots & \ddots & \vdots \\ {{\text{r}}_{{{\text{n1}}}} } & {{\text{r}}_{{{\text{n2}}}} } & \cdots & {{\text{r}}_{{{\text{nm}}}} } \\ \end{array} } \right]$$
  In the equation, "$\circ$" represents the fuzzy operator.
7. Fuzzy operator
  In the process of fuzzy transformation, fuzzy operators generally include primary factor determination type, primary factor prominence type, unbalanced average type and weighted average type, among others. The weighted average operator is characterized by clear weighting effects and high completeness. Therefore, this article uses the weighted average type operator for calculation. The specific calculation is as follows:
  15 $$b_{j} = \sum\limits_{i = 1}^{n} {a_{i} r_{ij} }, \quad j = 1,2, \ldots ,m$$
8. Handling evaluation results
  After the calculation process of comprehensive fuzzy evaluation, the final evaluation result “B” is obtained. At this stage it is necessary to process the assessment indicators. This article uses the maximum membership degree principle to process the fuzzy, comprehensive evaluation results and derive explicit evaluation results. The specific calculation method for the maximum membership degree principle is as follows:
  16 $${\text{b}}_{{{\text{i0}}}} = {\text{max}}\left\{ {{\text{b}}_{{\text{i}}} } \right\},1 \le i \le m$$
  Then the comprehensive assessment results of ${\text{i}}_{0}$ levels are determined. This operating method is relatively straightforward, with the majority of comprehensive evaluation approaches typically employing the maximum membership degree principle.
9. Multi-level fuzzy comprehensive evaluation
  Typically, when evaluating a complex system, it's necessary to consider the influences of various factors, which may also include sub-factors. Therefore, a comprehensive assessment of membership degrees across different factor levels is needed. In such cases, a multi-level assessment must be conducted in conjunction with the situation of each factor layer. When there are numerous influencing factors affecting the evaluation object, it is difficult to meaningfully assign the weights, which means that it is difficult to determine the hierarchy of individual factors within the overall assessment. In such situations, a multi-level fuzzy comprehensive assessment method is needed for determination.
  For example, when assessing the condition of a bridge, a bridge is divided into superstructure, substructure, auxiliary structure and bridge deck system according to its structure. Each structure is first subjected to a comprehensive assessment, and the assessment results then serve as single-factor assessments at a higher level. The weights of these four structures are denoted by A, and a comprehensive second-level fuzzy evaluation is performed. The calculation process is as follows.
  17 $$C = A \circ B = A \circ \left[ {\begin{array}{*{20}c} {B_{1} } \\ {B_{2} } \\ \vdots \\ {B_{k} } \\ \end{array} } \right] = A \circ \left[ {\begin{array}{*{20}c} {A_{1} } & {R_{1} } \\ {A_{2} } & {R_{2} } \\ \vdots & \vdots \\ {A_{K} } & {R_{k} } \\ \end{array} } \right]$$
  In the above equation, “C” represents the comprehensive evaluation result of the bridge condition. In cases with multiple influencing factors, it's advisable to first stratify and classify the factors, and then proceed with multi-level fuzzy comprehensive evaluation.

### Improved AHP-fuzzy comprehensive evaluation model for single tower steel box girder suspension bridges over the sea

**Figure 2:**
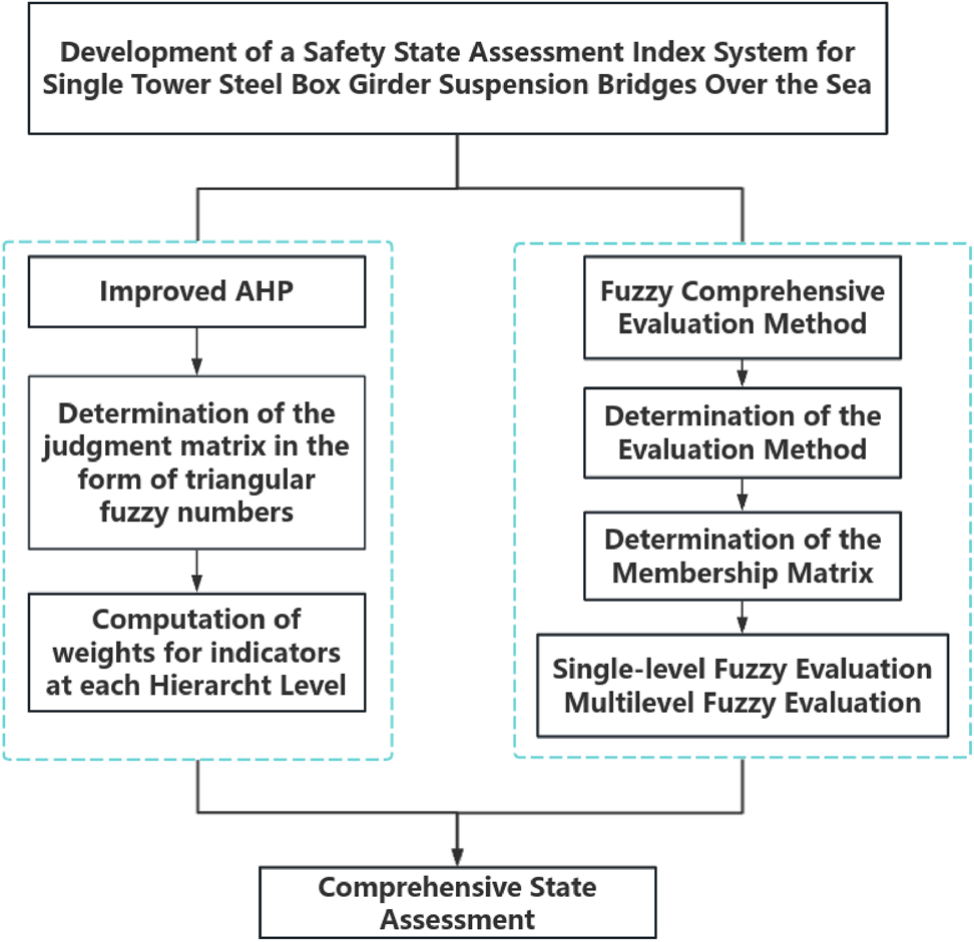
Schematic diagram of the evaluation model for single-tower steel box girder suspension bridges over the sea based on the improved AHP-fuzzy comprehensive evaluation.

The safety evaluation of single-tower steel box girder bridges over the sea includes various factors, including the steel box girder, concrete main tower, main cables, suspension rods and others, making it a typical multi-dimensional evaluation challenge. In the improved AHP method, although there are weights for each indicator, there is still a subjective element in the expert evaluation process. Therefore, it is crucial to further improve the quality of quantitative assessment through comprehensive fuzzy assessment methods. The evaluation model for single-tower steel box girder oversea suspension bridges based on the improved AHP-Fuzzy Comprehensive Evaluation is shown in Fig. 2.

## Health monitoring of a cross-sea single tower steel box girder suspension bridge

The condition monitoring of steel box girder suspension bridges with a tower over the sea primarily requires the installation of various types of sensors on site. These sensors collect monitoring data that reflects the structural safety status. By analyzing and processing this monitoring data, the health status of the structure is determined. This process creates a solid foundation for conducting bridge safety assessments and provides reference and decision support for bridge maintenance and management.

### Overview of the bridge health monitoring system

**Figure 3:**
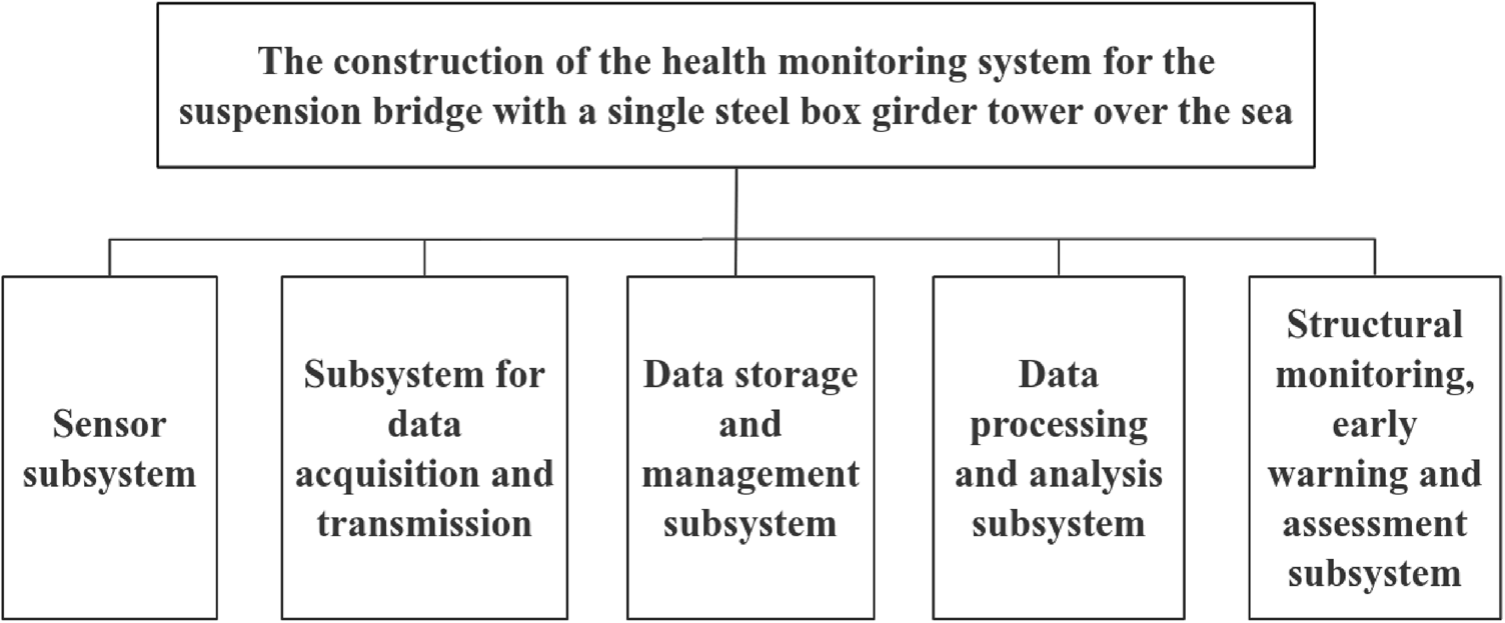
Structure of the health monitoring system of a single tower steel box girder suspension bridge over the sea.

The condition monitoring system for the steel box girder tower suspension bridge over the sea consists of five main subsystems: the sensor subsystem, the data acquisition and transmission subsystem, the data storage and management subsystem, the data processing and analysis subsystem and the structure monitoring. Early warning and security assessment subsystem. These subsystems are integrated using system integration technologies to coordinate the operation of hardware and software components. The configuration of the bridge condition monitoring system is shown in Fig. 3.

### Bridge health monitoring project and sensor placement

**Figure 4:**
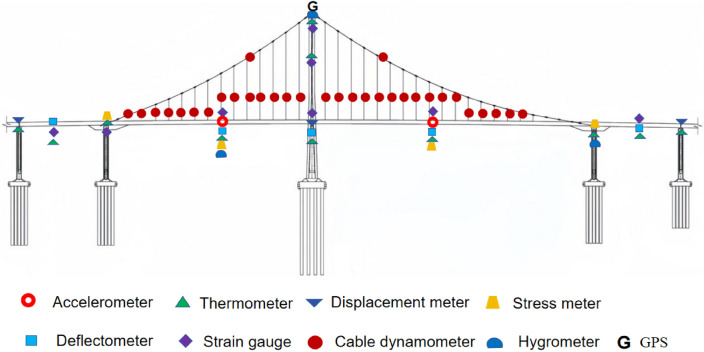
Schematic diagram of the monitoring point layout.

According to the structural characteristics of the 2nd Canal Bridge and taking into account the traffic volume and investment scale, the monitoring system for the 2nd Canal Bridge includes the following monitoring projects: wind speed and direction, structure temperature, deflection, cable saddle displacement, temperature and humidity, cable forces, anchor displacement, ship collision seismicity, preload force, cable clamping and vibration. The arrangement of the sensors is shown in Fig. 4, and a summary of the measurement points can be found in Table 2. The sampling frequency, units and data volume for each monitoring indicator are shown in Table 3.

**Table 2:** Monitoring content and number of measuring points.

Location	Monitoring project	Number of measuring points
Tower top, mid-span	Wind speed and direction	1
Cable saddle, tower top, mid-span	Temperature and humidity	2
Pier cap	Ship collision and seismicity	3
Below cable saddle	Concrete strain	20
Tower top	Tower deviation (GPS)	2
Critical section of the main beam	Main cable displacement (GPS)	1
Suspension cables	Cable forces	12
Steel box, main cable	Vibration	11
Expansion joint, anchor block	Displacement	6
Main girder section	Deflection	8
Main girder section of the bridge tower	Temperature	8

**Table 3:** Configuration of safety monitoring parameter acquisition.

Sensor type	Sampling frequency	Unit	Daily data volume
Strain measurement point	20 Hz	$\mathrm{\mu \varepsilon }$	1,728,000
Acceleration measurement point	10 Hz	mm/s^2^	864,000
Deflection measurement point	10 min/time	mm	144
Temperature measurement point	15 min/time	°C	96
Total daily data volume for the entire bridge			Approximately260,000

## Validation of engineering cases

### Project overview

**Figure 5:**
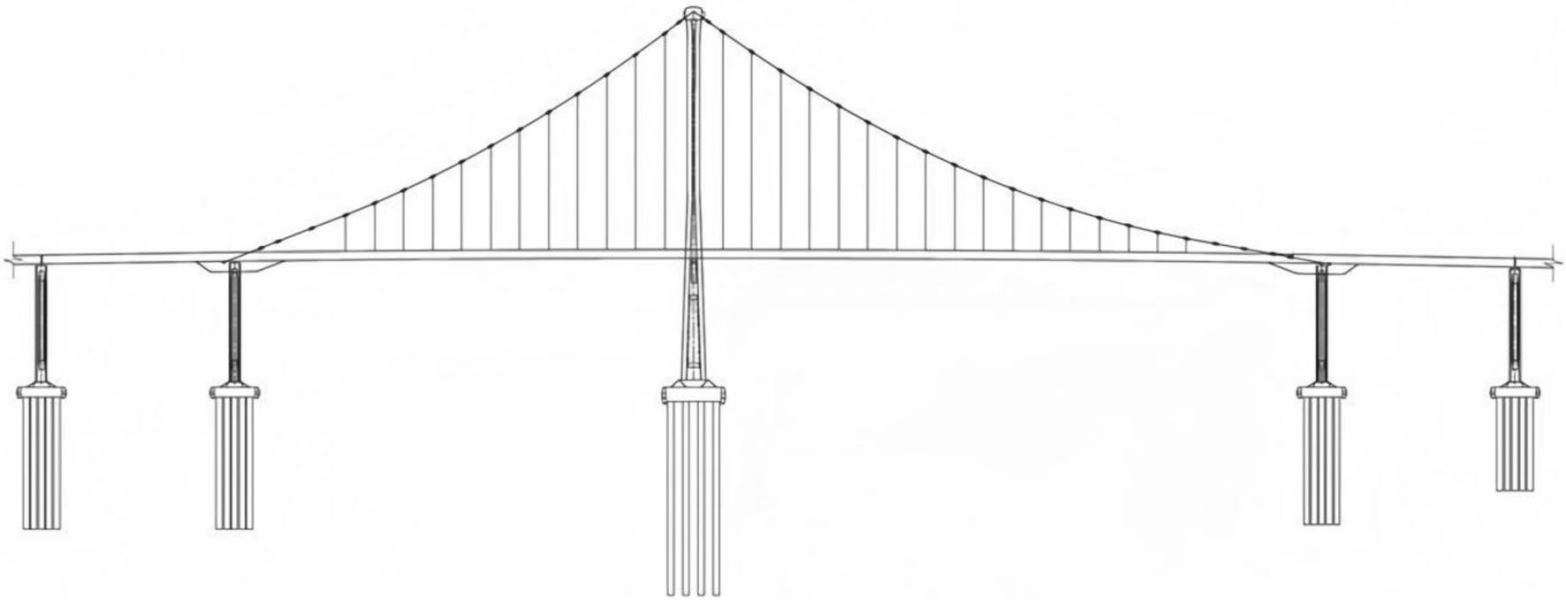
General structure of the bridge.

Bridge No. 2 is an important part of a northern coastal bridge and serves as an important sea connection between the eastern and western parts of the Bay City. It plays an important role in the Qinglan Expressway network. Bridge No. 2 is designed as a continuous, self-anchored steel box girder suspension bridge with a tower and a main span of 260 m. It is equipped with two main cables and 58 hanging rods. The span is 80 + 190 + 260 + 80 m with a total length of 610 m. The main and side panels utilize a suspension design with a continuous, semi-floating four panel system, as shown in Fig. 5. The tower of Bridge No. 2 consists of a single-column concrete tower and the main girder is made of segmented steel box girder construction. Both the main and side spans are configured as suspension systems, with a main span aspect ratio of 1/12.53 and a side span aspect ratio of 1/18.04.

### Application of the improved AHP in Bridge No. 2

#### Structure of the evaluation index system

Based on the structural form, characteristics and monitored content of Bridge No. 2, the AHP was used to hierarchize the structural system of Bridge No. 2. This led to the creation of a rating index system with corresponding hierarchical divisions. The highest level, the target level, refers to the comprehensive assessment of the safety status of Bridge No. 2. The middle level consists of primary indicators, a total of 8, and the lowest level includes secondary indicators, a total of 29. The hierarchical assessment The system for Bridge No. 2 is listed in Table 4.

**Table 4:** Improved AHP evaluation index system for Bridge No. 2.

Top level	Intermediate level	Bottom level
A comprehensive status assessment of No. 2 fairway bridge	B1. Steel box girder	C1. Main girder deflection
		C2. Main girder stress
		C3. Main girder lateral displacement
		C4. Main girder longitudinal displacement
		C5. Vibration frequency
	B2. Concrete main tower	C6. Main tower stress
		C7. Main tower longitudinal displacement
		C8. Main tower lateral displacement
	B3. Main cable system	C9. Main cable tension
		C10. Main cable protective layer
		C11. Cable clamp force
		C12. Cable saddle displacement
	B4. Suspender system	C13. Suspender cable tension
		C14. Suspender cable protective layer
		C15. Damping device
	B5. Anchor block	C16. Anchor block displacement
		C17. Concrete strength
	B6. Substructure	C18. Bearing displacement
		C19. Foundation settlement
		C20. Concrete strength
	B7. Auxiliary facilities	C21. Bridge deck pavement
		C22. Expansion joint
		C23. Drainage system
		C24. Lighting system
		C25. Railings
	B8. Environmental factors	C26. Temperature
		C27. Humidity
		C28. Wind direction and speed
		C29. Chloride ion concentration

In order to obtain the evaluation matrix for each indicator level of the suspension bridge, a survey questionnaire is developed, which is based on the created evaluation indicator system for Bridge No.2 and involves the 9-stage quantitative method for establishing evaluation criteria for each indicator, determining the hierarchical relationships and weight comparisons between the Indicators. Surveys on the No.2 Bridge Evaluation Indicator System were distributed to experts or scientists familiar with suspension bridge designs, and then promptly collected and analyzed.

#### Construction of an assessment matrix in the form of triangular fuzzy numbers for primary indicators and weight calculation

Based on the ratings assigned from the expert survey questionnaires, coupled with the finite element model analysis of Bridge No. 2, various monitoring values from the health monitoring system and with reference to the “Technical Condition Assessment Standards for Highway Bridges” (JTG/TH21-2011) a comprehensive calculation results in the assessment matrix shown in Table 5.

**Table 5:** Judgment matrix in the form of triangular fuzzy numbers for primary indicators.

	Steel box girder	Concrete main tower	Main cable system	Suspender system	Anchor block	Substructure	Auxiliary facilities	Environmental factors
Steel box Girder	[1,1,1]	[1,1,1]	[1/5,1/3,1/2]	[1/3,1/2,1]	[1/3,1/2,1]	[1,2,3]	[1,3,5]	[1,3,5]
Concrete Main Tower	[1,1,1]	[1,1,1]	[1/5,1/3,1/2]	[1/3,1/2,1]	[1/3,1/2,1]	[1,2,3]	[1,3,5]	[1,3,5]
Main cable System	[2,3,5]	[2,3,5]	[1,1,1]	[1,2,3]	[1,2,3]	[6,7,8]	[7,8,9]	[7,8,9]
Suspender System	[1,2,3]	[1,2,3]	[1/3,1/2,1]	[1,1,1]	[1,1,1]	[3,4,5]	[5,6,7]	[5,6,7]
Anchor block	[1,2,3]	[1,2,3]	[1/3,1/2,1]	[1,1,1]	[1,1,1]	[3,4,5]	[5,6,7]	[5,6,7]
Substructure	[1/3,1/2,1]	[1/3,1/2,1]	[1/8,1/7,1/6]	[1/5,1/4,1/3]	[1/5,1/4,1/3]	[1,1,1]	[2,3,5]	[2,3,5]
Auxiliary Facilities	[1/5,1/3,1]	[1/5,1/3,1]	[1/9,1/8,1/7]	[1/7,1/6,1/5]	[1/7,1/6,1/5]	[1/5,1/3,1/2]	[1,1,1]	[1,1,1]
Environmental Factors	[1/5,1/3,1]	[1/5,1/3,1]	[1/9,1/8,1/7]	[1/7,1/6,1/5]	[1/7,1/6,1/5]	[1/5,1/3,1/2]	[1,1,1]	[1,1,1]

1. The assessment matrix is subjected to a consistency approximation in order to obtain a consistency judgment matrix:

**Table:** 

	Steel box girder	Concrete main tower	Main cable system	Suspender system	Anchor block	Substructure	Auxiliary facilities	Environmental factors
Steel box girder	1	1	0.3389	0.5556	0.5556	2	3	3
Concrete main Tower	1	1	0.3389	0.5556	0.5556	2	3	3
Main cable System	3.1667	3.1667	1	2	2	7	8	9
Suspender system	2	2	0.5556	1	1	4	9.1667	9.1667
Anchor block	2	2	0.5556	1	1	4	9.1667	9.1667
Substructure	0.5556	0.5556	0.1438	0.2556	0.2556	1	3.1667	3.1667
Auxiliary Facilities	0.4222	0.4222	0.1257	0.1653	0.1653	0.3389	1	1
Environmental Factors	0.4222	0.4222	0.1257	0.1653	0.1653	0.3389	1	1
2. Calculate the nth root of the product of the elements in each row of the consistency judgment matrix:
  $$\begin{gathered} \hfill \\ \left[ {\begin{array}{*{20}c} 1 & 1 & {0.3389} & {0.5556} & {0.5556} & 2 & 3 & 3 \\ 1 & 1 & {0.3389} & {0.5556} & {0.5556} & 2 & 3 & 3 \\ {3.1667} & {3.1667} & 1 & 2 & 2 & 7 & 8 & 9 \\ 2 & 2 & {0.5556} & 1 & 1 & 4 & {9.1667} & {9.1667} \\ 2 & 2 & {0.5556} & 1 & 1 & 4 & {9.1667} & {9.1667} \\ {0.5556} & {0.5556} & {0.1438} & {0.2556} & {0.2556} & 1 & {3.1667} & {3.1667} \\ {0.4222} & {0.4222} & {0.1257} & {0.1653} & {0.1653} & {0.3389} & 1 & 1 \\ {0.4222} & {0.4222} & {0.1257} & {0.1653} & {0.1653} & {0.3389} & 1 & 1 \\ \end{array} } \right] \hfill \\ \downarrow \hfill \\ \left[ {\begin{array}{*{20}c} {1.0821} \\ {1.0821} \\ {3.4531} \\ {2.2865} \\ {2.2865} \\ {0.6427} \\ {0.1152} \\ {0.1152} \\ \end{array} } \right] \hfill \\ \end{gathered}$$
3. The above calculation results are orthogonalized to obtain the weight coefficient of each evaluation index: $W_{i} = \frac{{\overline{{W_{i} }} }}{{\sum\nolimits_{j = 1}^{n} {w_{j} } }}$
  $$\left[ {\begin{array}{*{20}c} {1.0821} \\ {1.0821} \\ {3.4531} \\ {2.2865} \\ {2.2865} \\ {0.6427} \\ {0.1152} \\ {0.1152} \\ \end{array} } \right]\mathop{\longrightarrow}\limits^{{{\text{Orthogonalization}}}}\left[ {\begin{array}{*{20}c} {0.0978} \\ {0.0978} \\ {0.3121} \\ {0.2067} \\ {0.2067} \\ {0.0581} \\ {0.0104} \\ {0.0104} \\ \end{array} } \right]$$
  Through the above calculation, the weights of the first level index steel box beam, concrete main tower, main cable system, suspension system, anchor bar, substructure, auxiliary facilities and environmental factors can be found as follows: 0.0978, 0.0978, 0, 3121, 0.2067, 0.2067, 0.0581, 0.0104, 0.0104, which shows that the weight value of the main cable is the largest and the weight value of the auxiliary facilities and environmental factors is the smallest.

#### Calculation of secondary indicator weights

1. Calculation of the secondary indicator weights for the primary evaluation criteria of the first-level box girder
  The method of constructing the judgment matrix in the triangular fuzzy number form for same-level indicators is consistent, and the comprehensive results are presented in the following matrix, as shown in Table 6.

**Table 6:** Triangular fuzzy number shape evaluation matrix for secondary indicators corresponding to the box girder evaluation criteria.

	Main girder deflection	Main girder stress	Main girder lateral displacement	Main girder longitudinal displacement	Vibration frequency
Main girder deflection	[1,1,1]	[1,2,3]	[1,1,1]	[1,2,3]	[1/3,1/2,1]
Main girder stress	[1/3,1/2,1]	[1,1,1]	[1/3,1/2,1]	[1,1,1]	[1/3,1/2,1]
Main girder lateral Displacement	[1,1,1]	[1,2,3]	[1,1,1]	[1,2,3]	[1/4,1/3,1/2]
Main girder longitudinal Displacement	[1/3,1/2,1]	[1,1,1]	[1/3,1/2,1]	[1,1,1]	[1/3,1/2,1]
Vibration frequency	[1,2,3]	[1,2,3]	[2,3,4]	[1,2,3]	[1,1,1]
  Similarly, the weights for the primary box girder evaluation criteria corresponding to main girder deflection, main girder stress, main girder lateral displacement, main girder longitudinal displacement, and vibration frequency can be determined. The results are shown in Table 7.

**Table 7:** Weight values for box girder evaluation criteria.

Evaluation criteria	Main girder deflection	Main girder stress	Main girder lateral displacement	Main girder longitudinal displacement	Vibration frequency
Weight values	0.2183	0.1225	0.1988	0.1225	0.3379
  According to Table 7, it can be observed that the weight value for the vibration frequency of the box girder is the highest, whereas the weight values for main girder stress and main girder longitudinal displacement are the lowest.
2. Calculation of secondary criterion weights corresponding to the primary evaluation criteria for a concrete main tower
  The method of constructing the judgment matrix in the triangular fuzzy number form for same-level indicators is consistent, and the comprehensive results are presented in the following matrix, as shown in Table 8.

**Table 8:** Triangular fuzzy number decision matrix for secondary criteria corresponding to evaluation criteria of concrete main tower.

	Main tower stress	Main tower longitudinal displacement	Main tower lateral displacement
Main tower stress	[1,1,1]	[1/5,1/4,1/3]	[1/3,1/2,1]
Main tower longitudinal displacement	[3,4,5]	[1,1,1]	[1,2,3]
Main tower lateral displacement	[1,2,3]	[1/3,1/2,1]	[1,1,1]
  Similarly, the weight values for the primary evaluation criteria corresponding to main tower stress, main tower longitudinal displacement, and main tower lateral displacement can be obtained, as shown in Table 9.

**Table 9:** Weight values of evaluation criteria for concrete main tower.

Evaluation criteria	Main tower stress	Main tower longitudinal displacement	Main tower lateral displacement
Weight values	0.1428	0.5721	0.2851
  From Table 9, it can be inferred that the weight assigned to the longitudinal displacement of the main tower is the highest, while the weight for the stress on the main tower is the lowest.
3. Calculation of secondary criterion weights corresponding to primary evaluation criteria for primary cable system
  The method of constructing the judgment matrix in the triangular fuzzy number form for same-level indicators is consistent, and the comprehensive results are presented in the following matrix, as shown in Table 10.

**Table 10:** Triangular fuzzy number decision matrix for secondary criteria corresponding to evaluation criteria of the main cable system.

	Main cable tension	Main cable protective layer	Cable clamp force	Cable saddle displacement
Main cable tension	[1,1,1]	[5,6,7]	[3,4,5]	[2,3,4]
Main cable protective layer	[1/7,1/6,1/5]	[1,1,1]	[1/3,1/2,1]	[1/4,1/3,1/2]
Cable clamp force	[1/5,1/4,1/3]	[4,5,6]	[1,1,1]	[1/3,1/2,1]
Cable saddle displacement	[1/4,1/3,1/2]	[2,3,4]	[2,3,4]	[1,1,1]
  Similarly, the weight values for the primary evaluation criteria can be determined according to the main cable force, main cable protection layer, clamping force and saddle displacement, as shown in Table 11.

**Table 11:** Weight values for evaluation criteria of the main cable system.

Evaluation criteria	Main cable tension	Main cable protective layer	Cable clamp force	Cable saddle displacement
Weight values	0.5576	0.0779	0.1356	0.2289
  From Table 11, it can be observed that the weight value for the main cable force is the highest, while the weight value for the clamp force is the smallest.
4. Calculation of secondary indicator weights corresponding to primary suspension rod system evaluation criteria
  The method of constructing the judgment matrix in the triangular fuzzy number form for same-level indicators is consistent, and the comprehensive results are presented in the following matrix, as shown in Table 12.

**Table 12:** Triangular fuzzy number form judgment matrix for secondary indicators corresponding to suspension rod system evaluation criteria.

	Suspender cable tension	Suspender cable protective layer	Damping device
Suspender cable tension	[1,1,1]	[4,5,6]	[2,3,4]
Suspender cable protective layer	[1/6,1/5,1/4]	[1,1,1]	[1/3,1/2,1]
Damping device	[1/4,1/3,1/2]	[1,2,3]	[1,1,1]
  Similarly, the weights for the evaluation criteria of the primary suspension rod system can be calculated according to the suspension rod tension, the suspension rod protective layer and the damper, as shown in Table 13.

**Table 13:** Suspension rod system evaluation criteria weights.

Evaluation criteria	Suspender cable tension	Suspender cable protective layer	Damping device
Weight values	0.5486	0.1223	0.3291
  From Table 13, it can be seen that the weight value for the tension of the suspender cable is the highest, while the weight value for the protective layer of the suspender cable is the lowest.
5. Calculation of weights for secondary indicators that correspond to the primary anchoring evaluation criteria.
  The method of constructing the judgment matrix in the triangular fuzzy number form for same-level indicators is consistent, and the comprehensive results are presented in the following matrix, as shown in Table 14.

**Table 14:** Triangular fuzzy number form judgment matrix for secondary indicators meeting the anchorage assessment criteria.

	Anchor block displacement	Concrete strength
Anchor block displacement	[1,1,1]	[3,4,5]
Concrete strength	[1/5,1/4,1/3]	[1,1,1]
  Similarly, the weight values for the displacement of the primary anchoring system and the concrete strength evaluation criteria can be calculated as shown in Table 15.

**Table 15:** Anchorage assessment criteria weighting values.

Evaluation criteria	Anchor block displacement	Concrete strength
Weight values	0.8000	0.2000
  From Table 15, it can be concluded that the weight value for anchor displacement is the highest, while the weight value for concrete strength is the lowest.
6. Calculation of weight for secondary indicators corresponding to the primary evaluation criteria for the substructure.
  The method of constructing the judgment matrix in the triangular fuzzy number form for indicators at the same level is consistent, and the comprehensive results are presented in the following matrix, as shown in Table 16.

**Table 16:** Triangular fuzzy number form judgment matrix for secondary indicators corresponding to the substructure evaluation criteria.

	Bearing displacement	Foundation settlement	Concrete strength
Bearing displacement	[1,1,1]	[1/4,1/3,1/2]	[4,5,6]
Foundation settlement	[2,3,4]	[1,1,1]	[6,7,8]
Concrete strength	[1/6,1/5,1/4]	[1/8,1/7,1/6]	[1,1,1]
  Similarly, the weight values for the primary substructure assessment criteria corresponding to support displacement, foundation settlement, and concrete strength can be calculated, as shown in Table 17.

**Table 17:** Weight values for the evaluation criteria for substructures.

Evaluation criteria	Bearing displacement	Foundation settlement	Concrete strength
Weight values	0.2831	0.6421	0.0748
  From Table 17, it can be seen that the weighting value for foundation settlement is the largest, while the weighting value for concrete strength is the smallest.
7. Calculation of weighting values for secondary indicators that correspond to the primary assessment criteria for ancillary facilities.
  The method of constructing the judgment matrix in the triangular fuzzy number form for same-level indicators is consistent, and the comprehensive results are presented in the following matrix, as shown in Table 18.

**Table 18:** Triangular fuzzy number form judgment matrix for secondary indicators corresponding to the evaluation criteria for additional facilities.

	Bridge deck pavement	Expansion joint	Drainage system	Lighting system	Railings
Bridge deck pavement	[1,1,1]	[1,1,1]	[1,2,3]	[6,7,8]	[4,5,6]
Expansion joint	[1,1,1]	[1,1,1]	[1,2,3]	[6,7,8]	[4,5,6]
Drainage system	[1/3,1/2,1]	[1/3,1/2,1]	[1,1,1]	[2,3,4]	[2,3,4]
Lighting system	[1/8,1/7,1/6]	[1/8,1/7,1/6]	[1/4,1/3,1/2]	[1,1,1]	[1/3,1/2,1]
Railings	[1/6,1/5,1/4]	[1/6,1/5,1/4]	[1/4,1/3,1/2]	[1,2,3]	[1,1,1]
  Similarly, weight values can be calculated for the primary assessment criteria for ancillary facilities relating to bridge decking, expansion joints, drainage systems, lighting systems and railings, as shown in Table 19.

**Table 19:** Weighting values of the evaluation criteria for additional facilities.

Evaluation criteria	Bridge deck pavement	Expansion joint	Drainage system	Lighting system	Railings
Weight values	0.3512	0.3512	0.1781	0.0467	0.0710
  From Table 19, it can be seen that the weight values for bridge deck and expansion joints are the highest, while the weight value for railings is the lowest.
8. Calculation of weight values for secondary indicators that correspond to the assessment criteria for primary environmental factors.
  The method of constructing the judgment matrix in the triangular fuzzy number form for same-level indicators is consistent, and the comprehensive results are presented in the following matrix, as shown in Table 20.

**Table 20:** Triangular fuzzy number form decision matrix for secondary indicators corresponding to the environmental factor evaluation criteria.

	Wind direction and speed	Temperature	Humidity	Chloride ion concentration
Wind direction and speed	[1,1,1]	[2,3,4]	[3,4,5]	[1/3,1/2,1]
Temperature	[1/4,1/3,1/2]	[1,1,1]	[1,2,3]	[1/4,1/3,1/2]
Humidity	[1/5,1/4,1/3]	[1/3,1/2,1]	[1,1,1]	[1/5,1/4,1/3]
Chloride ion concentration	[1,2,3]	[2,3,4]	[3,4,5]	[1,1,1]
  Similarly, the weight values for wind speed, temperature and humidity can be determined, which correspond to the primary evaluation criteria for environmental factors. As shown in Table 21:

**Table 21:** Weighting values of the evaluation criteria for environmental factors.

Evaluation criteria	Wind direction and speed	Temperature	Humidity	Chloride ion concentration
Weight values	0.2641	0.1871	0.2072	0.3596
  From Table 21, it can be concluded that the weight value for CL ions is the highest, while the weight value for temperature is the lowest.

### Safety assessment of Canal Bridge No. 2 based on an improved comprehensive AHP + Fuzzy assessment

In accordance with the improved AHP applied to the evaluation criteria system for the components of Bridge No. 2, it is divided into three levels: the highest level (objective level), the intermediate level (first-level indicators), and the lowest level (second-level indicators). In this paper, the eight first-level indicators at the intermediate level are designated as the first layer of the factor set, denoted as $U_{1}$, and the 28 s-level indicators at the lowest level are designated as the second layer of the factor set, denoted as $U_{2}$, The weight values of each level's factors are determined based on the calculations presented in "Bridge health monitoring project and sensor placement" section of this paper.

Fuzzy statistical method is employed in this study to determine the membership functions. A survey questionnaire is distributed to relevant experts or scholars to individually evaluate and score all the factors in the third layer of factors set $U_{2}$. The recipients of the survey questionnaire include the users of Bridge No. 2, maintenance managers, and individuals involved in the bridge load testing. Fuzzy evaluations in this paper are primarily based on relevant specifications, combined with finite element simulation responses, actual data from health monitoring systems, and the real condition of the bridge. The fuzzy evaluations are classified into five levels: "Intact," "Good," "Fairly Good," "Poor," and "Dangerous," denoted as V1 = Intact, V2 = Good, V3 = Fairly Good, V4 = Poor, V5 = Dangerous. The set of fuzzy evaluations is represented as V = {Intact, Good, Fairly Good, Poor, Dangerous}.

Statistical analysis was performed on the distributed and collected expert questionnaires to determine the membership frequencies or membership degrees for each factor indicator. The statistical results are shown in Table 22.

**Table 22:** Statistical table of membership level of each index.

Top level	Intermediate level	Bottom level	Intact	Good	Fairly good	Poor	Dangerous
A. Bridge No. 2 comprehensive state assessment	B1. Steel box girder	C1. Main girder deflection	0.5	0.4	0.1	0	0
		C2. Main girder stress	0.5	0.4	0.1	0	0
		C3. Main girder lateral displacement	0.6	0.4	0	0	0
		C4. Main girder longitudinal displacement	0.5	0.4	0.1	0	0
		C5. Vibration frequency	0.8	0.2	0	0	0
	B2. Concrete main tower	C6. Main tower stress	0.6	0.4	0	0	0
		C7. Main tower longitudinal displacement	0.7	0.2	0.1	0	0
		C8. Main tower lateral displacement	0.9	0.1	0	0	0
	B3. Main cable system	C9. Main cable tension	0.8	0.1	0.1	0	0
		C10. Main cable protective layer	0.3	0.5	0.1	0.1	0
		C11. Cable clamp force	0.6	0.3	0.1	0	0
		C12. Cable saddle displacement	0.7	0.2	0.1	0	0
	B4. Suspender system	C13. Suspender cable tension	0.3	0.5	0.2	0	0
		C14. Suspender cable protective layer	0.3	0.3	0.3	0.1	0
		C15. Damping device	0.6	0.3	0.1	0	0
	B5. Anchor block	C16. Anchor block displacement	0.5	0.3	0.1	0.1	0
		C17. Concrete strength	0.5	05	0	0	0
	B6. Substructure	C18. Bearing displacement	0.3	0.6	0.1	0	0
		C19. Foundation settlement	0.6	0.2	0.2	0	0
		C20. Concrete strength	0.5	0.2	0.3	0	0
	B7. Auxiliary facilities	C21. Bridge deck pavement	0.1	0.4	0.4	0.1	0
		C22. Expansion joint	0.2	0.5	0.2	0.1	0
		C23. Drainage system	0	0.4	0.5	0.1	0
		C24. Lighting system	0.6	0.2	0.1	0.1	0
		C25. Railings	0.6	0.2	0.2	0	0
	B8. Environmental factors	C26. Temperature	0.2	0.5	0.3	0	0
		C27. Humidity	0.3	0.6	0.1	0	0
		C28. Wind direction and speed	0.5	0.4	0.1	0	0
		C29. Chloride ion concentration	0.4	0.5	0.1	0	0

#### Index evaluation of the primary index layer of the No. 2 Channel bridge

1. Evaluation of steel box girder indicator
  The fuzzy matrix corresponding to the indicators of the second level of the steel box girder is:
  $$R_{B1} = \left[ {\begin{array}{*{20}c} {C1} \\ {C2} \\ {C3} \\ {C4} \\ {C5} \\ \end{array} } \right] = \left[ {\begin{array}{*{20}c} {0.5} & {0.4} & {0.1} & 0 & 0 \\ {0.5} & {0.4} & {0.1} & 0 & 0 \\ {0.6} & {0.4} & 0 & 0 & 0 \\ {0.5} & {0.4} & {0.1} & 0 & 0 \\ {0.8} & {0.2} & 0 & 0 & 0 \\ \end{array} } \right]$$
  The weights of the second level indicators corresponding to the steel box girder criteria are as follows:
  $$\omega_{B1} = \left[ {\begin{array}{*{20}c} {0.2183} & {0.1225} & {0.1988} & {0.1225} & {0.3379} \\ \end{array} } \right]$$
  The degree of membership defined for the steel box girder indicators is:
  $$B_{1} = \omega_{B1} \times R_{B1} = \left[ {\begin{array}{*{20}c} {0.6213} & {0.3324} & {0.0463} & 0 & 0 \\ \end{array} } \right]$$
  According to the principle of maximum membership degree, the highest membership degree of 0.6213 is selected as the comprehensive evaluation result for the steel box girder indicators. Therefore, when assessing its indicators, it must be assumed that it is in an intact state.
2. Evaluation of the concrete main tower indicator
  The fuzzy matrix corresponding to the secondary indicators of the main concrete tower is:
  $$R_{B2} = \left[ {\begin{array}{*{20}c} {C6} \\ {C7} \\ {C8} \\ \end{array} } \right] = \left[ {\begin{array}{*{20}c} {0.6} & {0.4} & 0 & 0 & 0 \\ {0.7} & {0.2} & {0.1} & 0 & 0 \\ {0.9} & {0.1} & 0 & 0 & 0 \\ \end{array} } \right]$$
  The weights associated with the secondary indicators of the main concrete tower are:
  $$\omega_{B2} = \left[ {\begin{array}{*{20}c} {0.1428} & {0.5721} & {0.2851} \\ \end{array} } \right]$$
  The membership set for the primary indicators of the steel box girder main tower is:
  $$B_{2} = \omega_{B2} \times R_{B2} = \left[ {\begin{array}{*{20}c} {0.7427} & {0.2001} & {0.5721} & 0 & 0 \\ \end{array} } \right]$$
  According to the maximum membership degree principle, the highest membership degree of 0.7427 should be selected as the comprehensive evaluation result for the concrete main tower indicators to judge it as being in good condition.
3. Main cable system
  The fuzzy matrix corresponding to the secondary indicators of the main cable system is as follows:
  $$R_{B3} = \left[ {\begin{array}{*{20}c} {C9} \\ {C10} \\ {C11} \\ {C12} \\ \end{array} } \right] = \left[ {\begin{array}{*{20}c} {0.8} & {0.1} & {0.1} & 0 & 0 \\ {0.3} & {0.5} & {0.1} & {0.1} & 0 \\ {0.6} & {0.3} & {0.1} & 0 & 0 \\ {0.7} & {0.2} & {0.1} & 0 & 0 \\ \end{array} } \right]$$
  The weights of the secondary indicators corresponding to the main criteria of the cable system are as follows:
  $$\omega_{B3} = \left[ {\begin{array}{*{20}c} {0.5576} & {0.0779} & {0.1356} & {0.2289} \\ \end{array} } \right]$$
  The membership degree set for the main cable system indicators is as follows:
  $$B_{3} = \omega_{B3} \times R_{B3} = \left[ {\begin{array}{*{20}c} {0.7110} & {0.1812} & {0.1317} & {0.0779} & 0 \\ \end{array} } \right]$$
  According to the maximum membership degree principle, the highest membership degree of 0.7110 is selected as the comprehensive evaluation result for the main indicators of the cable system, and the system is judged to be in good condition.
4. Suspension rod system
  The fuzzy matrix corresponding to the secondary indicators of the suspension rod system is as follows:
  $$R_{B4} = \left[ {\begin{array}{*{20}c} {C13} \\ {C14} \\ {C15} \\ \end{array} } \right] = \left[ {\begin{array}{*{20}c} {0.3} & {0.5} & {0.2} & 0 & 0 \\ {0.3} & {0.3} & {0.3} & {0.1} & 0 \\ {0.6} & {0.3} & {0.1} & 0 & 0 \\ \end{array} } \right]$$
  The weights of the secondary indicators corresponding to the suspension rod system are as follows:
  $$\omega_{B4} = \left[ {\begin{array}{*{20}c} {0.5486} & {0.1223} & {0.3291} \\ \end{array} } \right]$$
  The degree of membership established for the indicators of the suspension rod system is as follows:
  $$B_{4} = \omega_{B4} \times R_{B4} = \left[ {\begin{array}{*{20}c} {0.3987} & {0.4097} & {0.1793} & {0.0123} & 0 \\ \end{array} } \right]$$
  According to the maximum membership degree principle, taking a maximum membership degree of 0.4097 as the comprehensive evaluation result for the suspension rod system indicators, the system should be judged to be in good condition.
5. Anchor block
  The fuzzy matrix corresponding to the secondary indicators of the anchor block is as follows:
  $$R_{B5} = \left[ {\begin{array}{*{20}c} {C16} \\ {C17} \\ \end{array} } \right] = \left[ {\begin{array}{*{20}c} {0.5} & {0.3} & {0.1} & {0.1} & 0 \\ {0.5} & {0.5} & 0 & 0 & 0 \\ \end{array} } \right]$$
  The weights of the secondary indicators that correspond to the anchor block criteria are as follows:
  $$\omega_{B5} = \left[ {\begin{array}{*{20}c} {0.8000} & {0.2000} \\ \end{array} } \right]$$
  The membership degree set for the anchor block criteria is as follows:
  $$B_{5} = \omega_{B5} \times R_{B5} = \left[ {\begin{array}{*{20}c} {0.5000} & {0.3400} & {0.8000} & 0 & 0 \\ \end{array} } \right]$$
  According to the maximum membership degree principle, and taking the maximum membership degree of 0.8000 as the comprehensive assessment result for the anchor block criteria, the indicators should be judged to be relatively good.
6. Substructure
  The fuzzy matrix corresponding to the secondary indicators of the substructure criteria is as follows:
  $$R_{B6} = \left[ {\begin{array}{*{20}c} {C18} \\ {C19} \\ {C20} \\ \end{array} } \right] = \left[ {\begin{array}{*{20}c} {0.3} & {0.6} & {0.1} & 0 & 0 \\ {0.6} & {0.2} & {0.2} & 0 & 0 \\ {0.5} & {0.2} & {0.3} & 0 & 0 \\ \end{array} } \right]$$
  The weights of the secondary indicators that correspond to the sub-structural criteria are:
  $$\omega_{B6} = \left[ {\begin{array}{*{20}c} {0.2821} & {0.6421} & {0.0748} \\ \end{array} } \right]$$
  The membership degree set for the substructure criteria is:
  $$B_{6} = \omega_{B6} \times R_{B6} = \left[ {\begin{array}{*{20}c} {0.5073} & {0.3126} & {0.1790} & 0 & 0 \\ \end{array} } \right]$$
  According to the maximum membership degree principle, taking the highest membership degree of 0.5073 as the comprehensive evaluation result for the substructure criteria, the indicators should be judged to be in a sound condition.
7. Auxiliary facilities
  The fuzzy matrix corresponding to the secondary indicators of the auxiliary facilities criteria is:
  $$R_{B7} = \left[ {\begin{array}{*{20}c} {C21} \\ {C22} \\ {C23} \\ {C24} \\ {C25} \\ \end{array} } \right] = \left[ {\begin{array}{*{20}c} {0.1} & {0.4} & {0.4} & {0.1} & 0 \\ {0.2} & {0.5} & {0.2} & {0.1} & 0 \\ 0 & {0.4} & {0.5} & {0.1} & 0 \\ {0.6} & {0.2} & {0.1} & {0.1} & 0 \\ {0.6} & {0.2} & {0.2} & 0 & 0 \\ \end{array} } \right]$$
  The weights of the secondary indicators corresponding to the criteria for auxiliary facilities are:
  $$\omega_{B7} = \left[ {\begin{array}{*{20}c} {0.3512} & {0.3512} & {0.1781} & {0.0467} & {0.0710} \\ \end{array} } \right]$$
  The membership degree set for the criteria for auxiliary facilities is:
  $$B_{7} = \omega_{B7} \times R_{B7} = \left[ {\begin{array}{*{20}c} {0.1066} & {0.4108} & {0.3186} & {0.0821} & 0 \\ \end{array} } \right]$$
  According to the maximum membership degree principle, selecting the highest membership degree of 0.4108 as the comprehensive assessment result for the aid facility criteria indicates that the indicators are in good condition.
8. Environmental factors
  The fuzzy matrix corresponding to the secondary indicators of the environmental factors criteria is:
  $$R_{B6} = \left[ {\begin{array}{*{20}c} {C26} \\ {C27} \\ {C28} \\ {C29} \\ \end{array} } \right] = \left[ {\begin{array}{*{20}c} {0.2} & {0.5} & {0.3} & 0 & 0 \\ {0.3} & {0.6} & {0.1} & 0 & 0 \\ {0.5} & {0.4} & {0.1} & 0 & 0 \\ {0.4} & {0.5} & {0.1} & 0 & 0 \\ \end{array} } \right]$$
  The weights of the secondary indicators corresponding to the criteria for environmental factors are:
  $$\omega_{B6} = \left[ {\begin{array}{*{20}c} {0.2641} & {0.1871} & {0.2072} & {0.3596} \\ \end{array} } \right]$$
  The degree of membership established for the environmental factors criteria is:
  $$B_{8} = \omega_{B8} \times R_{B8} = \left[ {\begin{array}{*{20}c} {0.3564} & {0.5070} & {0.1546} & 0 & 0 \\ \end{array} } \right]$$
  According to the maximum membership degree principle and choosing the highest membership degree of 0.5070 as the comprehensive evaluation result for the environmental factor criteria, the indicators should be evaluated as being in good condition.

#### Overall safety assessment of Bridge No. 2

The fuzzy matrix corresponding to the primary indicators of Bridge No. 2 is as follows:

$$R_{A} = \left[ {\begin{array}{*{20}c} {B_{1} } \\ {B_{2} } \\ {B_{3} } \\ {B_{4} } \\ {B_{5} } \\ {B_{6} } \\ {B_{7} } \\ {B_{8} } \\ \end{array} } \right] = \left[ {\begin{array}{*{20}c} {0.6213} & {0.3324} & {0.0463} & 0 & 0 \\ {0.7427} & {0.2001} & {0.5721} & 0 & 0 \\ {0.7110} & {0.1812} & {0.1317} & {0.0779} & 0 \\ {0.3987} & {0.4097} & {0.1793} & {0.0123} & 0 \\ {0.5000} & {0.3400} & {0.8000} & 0 & 0 \\ {0.5073} & {0.3126} & {0.1790} & 0 & 0 \\ {0.1066} & {0.4108} & {0.3186} & {0.0821} & 0 \\ {0.3564} & {0.5070} & {0.1546} & 0 & 0 \\ \end{array} } \right]$$

The weights of the primary indicators corresponding to Bridge No. 2 are:

$$\omega_{A} = \left[ {\begin{array}{*{20}c} {0.0978} & {0.0978} & {0.3121} & {0.2067} & {0.2067} & {0.0581} & {0.0104} & {0.0104} \\ \end{array} } \right]$$

The membership degree set for Bridge No. 2 is:

$$A = \omega_{A} \times R_{A} = \left[ {\begin{array}{*{20}c} {0.6413} & {0.3259} & {0.2065} & {0.0277} & 0 \\ \end{array} } \right]$$

According to the maximum membership degree principle, selecting the highest membership degree of 0.6413 as the result of the comprehensive safety assessment for Bridge No. 2 indicates that the overall safety assessment is in a solid state.

Based on the above, the comprehensive safety assessment results of various systems and the overall structure of Bridge No. 2 are shown in Table 23.

**Table 23:** Comprehensive safety assessment results of various systems and overall structure of Bridge No. 2.

	Bridge structural systems	Safety assessment results	Overall comprehensive assessment results
Bridge No. 2 comprehensive state assessment	Steel box girder	Intact	Intact
	Concrete main tower	Intact	
	Main cable system	Intact	
	Suspender system	Good	
	Anchor block	Fairly Good	
	Substructure	Intact	
	Auxiliary facilities	Good	
	Environmental factors	Good	

The safety status assessment of Bridge No. 2 relied on the improved AHP-fuzzy comprehensive evaluation method proposed in this study and showed favorable results. The introduced improved AHP-Fuzzy comprehensive evaluation method for bridge safety evaluation has certain significance for technical guidance.

## Conclusions

- Using the triangular fuzzy number method, improvements have been made to the judgment matrix, allowing experts to rate the importance of indicators without being confined to providing an exact numerical value; instead, they need only provide a score range. This reduces the influence of subjective factors on the evaluation results, ensures the consistency of the judgment matrix, and improves the performance of determining the AHP indicator weight.
- By combining the improved AHP with a comprehensive fuzzy assessment, a model is constructed to evaluate the safety status of a single-tower steel box girder suspension bridge over the sea. Building on the determination of the weights of various evaluation indicators using the improved AHP, the comprehensive fuzzy evaluation method is applied to calculate the membership degrees of each indicator, thereby evaluating the safety status of the bridge, resulting in a more reasonable and reliable evaluation result.
- The assessment of the safety status of the No. 2 Channel Bridge shows that the bridge is currently in good condition overall and should undergo routine maintenance in the future. It was found that the main cable system of the suspension bridge has the highest weight values, while the weightage of auxiliary facilities and environmental factors is the lowest. Among the environmental factors, chloride ions (CL) were assigned the highest weightage, which can corrode the concrete structure of the bridge, requiring increased additional anti-corrosion measures.
- The assessment of the safety status of the No. 2 Channel Bridge shows that the proposed method is effective in assessing bridges under the condition that data from health monitoring systems are collected, so as to determine the safety status of the bridge. This method also accurately evaluates the index system and is of considerable importance for engineering guidance.

## Data Availability

The datasets generated during and/or analysed during the current study are available from the corresponding author on reasonable request.
